# Care of the Teeth

**Published:** 1867-07

**Authors:** 


### CARE OF THE TEETH.

The causes of decay in the teeth may be divided into two classes, viz: direct and incidental. There are two concurrent conditions or influences that generally meet to produce the former, (that is direct decay). The first of these conditions is imperfect organization. The second is an unhealthy condition of the fluids of the mouth, or contact with some of the various acids that find their way into the Dental territory from time to time, under various pretexts. Though the mineral acids are much more energetic in their action, when brought in contact with the substance of the teeth, yet acetic acid, (which results from the fermentation of vegetable substances that contain a portion of sugar), (or starch.—Ed.) works by far the greater amount of mischief in the Dental organism.

A particle of food lodged about the necks of the teeth, and exposed to the moisture and temperature of the mouth, quickly undergoes fermentation, and the acid result instantly demands its equivalent of alkali from the contiguous teeth; for the spaces between the necks of the teeth being the most convenient receptacle for the lodgment of food, is the point where this kind of decay generally makes its attack.

Sometimes, from a vitiated condition of the fluids of the mouth, and a want of the necessary attention to cleanliness, particles of food are retained between the cheeks, or possibly between the tongue and teeth, till they undergo fermentation, and decomposition, with a like disastrous result.

Sometimes the fissures that mark the grinding surface of the molars, and bicuspids, and occasionally the lingual surfaces of the incisors, divide the enamel entirely, leaving an opening by which the saccharine fluids of the mouth are admitted to contact with the bone of the teeth, thus furnishing the conditions and materials where another chemical laboratory may be run with ruinous results.

This accident of imperfect enamel furnishes almost the only condition under which decay of the teeth is likely to occur, when proper, timely attention might not prevent it, especially where the teeth possess a sound healthy constitution in other respects.

True, the teeth of children frequently decay before they could reasonably be expected to exercise the forethought and skill necessary to prevent it. In this case the responsibility justly falls on parents, who in too many instances are about as incompetent as the children.

How many among us run through the whole course of rational intelligent existence, and never occupy any higher range of mental or moral activity, never entertain any more lofty aspirations, than to know the price of stocks or produce, or the latest Paris fashions? How many are content to remain in the most profound and hopeless ignorance of the laws of the Creator in reference to their physical well-being, while their whole aim and effort is circumscribed by the narrow limits and transient interests of the present life? We will not pursue this digression further at present, than to say that this same blind, stupid, contented ignorance is justly chargeable with the loss of a large proportion of all the teeth that are lost from decay.

We said, on a former occasion, that chemical affinity was the immediate, active, efficient agent in decomposing the substance of the teeth. This is strictly true; but such chemical affinity is only active under certain conditions of warmth, moisture, etc., and the presence of an acid or some fermentable substance whose acid reaction is capable of decomposing the substance of the teeth, (and then fermentation is accomplished, and the acid is produced before the substance of the teeth is attacked or destroyed.)

And a sufficient time for the destruction of the tooth is essential; for these operations are not carried forward with the same degree of rapidity that marks chemical action when explosive substances are brought within the range of mutual chemical influences; but, on the contrary, quite gradually, being retarded in their action, suspended, or perhaps altogether arrested by a change of surrounding conditions.

Of the incidental or indirect causes of decay in the Dental family, the range is much greater. Whatever impairs the general health while the teeth are in process of formation, affects them injuriously. Whatever curtails or interrupts the osseous development, crowds or compresses the teeth into a space too small to accommodate them all in proper position. Thus, we frequently see persons whose teeth are lapped or crossed in their arrangement, thereby affording a convenient place for the lodgment of food, and thereby effecting decay of the teeth.

General derangement of the health, even after the teeth are formed, if continued for a length of time, is certain to affect the teeth injuriously. Chills, dyspepsia, hypochondria, of long standing, especially if made the occasion for active medication, are certain to injure the teeth. Any overwrought mental excitement, such as home-sickness, dread of impending evil, or hopeless sorrow, if indulged in for a length of time, will surely injure the teeth in the end. Whatever undermines the constitution, preys upon the general health, depresses the spirits, or weakens the vital force, invades the sanctity of the teeth in an indirect manner perhaps, but never fails to reach the end where sufficient time is afforded. In the language employed by the founders of our federal institutions, we may truly say of the different members of the physical system, "*united* we stand, *divided* we fall." It is not necessary to specify all the causes that may effect the teeth incidentally. In a word, they are as numerous and diversified as the evil influences that surround us on every hand, and at all times stand ready to overwhelm frail and erring humanity.

But, however modified or diversified the incidental causes of decay in the teeth may be, the direct and immediate cause is chemical affinity. This brings us round to the point from which we set out.

The means and methods by which the teeth may be guarded against these complex and diversified evil influences, may be stated in as concise and explicit terms as the immediate and direct cause or agency by which the mischief is wrought, viz: Cleanliness—constant, absolute, scrupulous cleanliness.

By cleanliness, we understand the entire absence of, or freedom from, dirt of all kinds. Probably no lexicographer has ever given a better definition of the word "dirt" than a professor in a Southern college, who told his class that the word dirt was not the name of any one object or class of objects, unconditionally, but applied to any and every thing that might chance to be found in the wrong place. Thus the soil is altogether proper in the field or garden, is the true basis of real estate, the area in which all agricultural operations are performed, the source whence our daily food is derived; but the moment it is found on our carpet or clothing, on our hands or face, it immediately becomes dirt, and we can make nothing else of it, only by returning it to its proper place.

And there is yet another peculiarity that it becomes necessary for us to notice in this connection. There are instances in which objects change their character, in this respect, with the change of time and circumstances. For instance, a professor of the culinary art may have the cleanest hands in the world, while preparing bread for the oven, yet at the same time they are covered with paste and flour. There is nothing improper or offensive in this while the operation lasts, but the moment it is finished, without any additional deposit on these same hands, they instantly become dirty, and so remain till the last particles of flour, paste, shortening and sweetening are removed.

And the necessity for this cleansing process prevails throughout every department of human interest, during all time. Not only our hands and teeth, but our ecclesiastical and political institutions, our fields and highways, our houses and clothing, the food that we eat, the most secret thought that finds a lodgement in our mind, or the most subtle emotion that stirs our inmost nature, demand constant, persistent, intelligent purification.

Indeed, this cleansing process appears to have formed a distinct and prominent feature in the design of the all-wise Creator, when he planned this vast and complicated terrestial fabric. Storms purge away from the atmosphere the pestilential and poisonous vapors that would otherwise destroy animal life. Pestilence thins over-crowded, and, consequently, excessively filthy communities. The Asiatic Cholera has been styled as chief of staff on the Almighty's sanitary commission. Throughout the entire range of human observation this purifying process is continually going on both in communities and individuals, and whenever offenders are permitted to go "unwhipt of justice," wherever injustice or impertinence goes unrebuked, where any wrong remains unrighted, where an individual abandons himself to the control of any vicious habits, or sits down content to let matters take their course, right or wrong, there we mark the advancing footsteps of decay and death.

The first and most important, among the means and methods by which the teeth may be kept in a clean and healthy condition, is exercise or use—the mastication of an amount of food appropriately selected and prepared to meet the wants of the organization or individual to which they pertain. Perhaps nothing more than this would be necessary to secure the health of the teeth in an individual whose present health was perfect, whose habits were unexceptionable, whose physical constitution, even to the arrangement of the teeth, was faultless, whose intellectual development was all that the Creator intended, and whose moral and social relations were only sources of hope, without a doubt or fear, and happiness without alloy. But where shall we find the individual thus favorably circumstanced and constituted? We ask, and pause for a reply.

Our ignorance of a fact does not suppress its reality—does not annihilate it. Ignorance of danger has no power to elude its pursuit, or parry its attack. In a word, ignorance on any point is weakness,—invites and facilitates the approach of danger, and embarrasses the efforts of those who would seek to secure their own safety.

It is nothing unusual that persons possessing a fair share of intelligence in ordinary affairs, but who have not bestowed any especial care on the preservation of their teeth, suppose them all to be perfectly sound, until decay has effected a breach in the "citadel of life" of one or more of these organs. The first condition to success in any enterprise, is a full and clear understanding of the whole matter in all its relations and dependencies.

When the teeth are to be cleansed, it is important that we first know whether they are decayed, whether they require to be freed from deposits of tartar, or whether it is only necessary to brush away the fragments of our last meal that may have been left about their necks while eating.

A good elastic brush, or tooth-pick, or both together, followed by a mouthful of water, forced between the teeth by the motion of the lips, cheeks, etc., are all that will be required where nothing more is necessary than to remove the fragments of food from sound and healthy teeth and gums. But a brush and pick are altogether inadequate to the removal of tartar, where it has remained long enough to become hard, though nothing more would be necessary while the deposits remain soft.

In favorable cases, a few moments every day employed in well-directed attention to the teeth, would be quite sufficient to secure them in the desired condition. There are other cases which, if found in the mouth of the most skillful and competent Dentist, would tax his skill, patience and perseverance to the utmost, together with the highest professional aid he could procure, to bring about the desired conditions.

If the teeth are foul with deposits of tartar, several steel instruments, triangular, sharp, curved or straight, will be required for its removal, and like all other operations requiring some degree of handicraft skill, it is more readily, more comfortably, and more effectually accomplished by a hand trained to the performance of kindred tasks; though any individual having good use of his hands, and provided with the instruments that are necessary, and endowed with a due share of patient, intelligent perseverance, may practice on his own teeth with advantage, especially in simple cases where the disease has not progressed too far. The greatest danger to be apprehended from this home treatment arises from the fact that inefficient or misdirected efforts may lull the apprehension of the patient into a fancied security, cause him to relax his vigilance or suspend his efforts, and thus permit the disease to run its course, and thus accomplish the destruction of the teeth. For there is no disease to which the teeth are liable that will more certainly destroy them than this, when permitted to take its natural course.

There is one more condition to success, and that is, determined, persistent, intelligent effort under the guidance of enlightened judgment.

While ignorant effort exhausts itself in bootless strife to accomplish something beyond its reach, the most complete, thorough and explicit knowledge is unprofitable and vain until it assumes the guidance of earnest, patient labor.

In the common affairs of life it is hard to say which is the most prolific source of failure, ignorance or indolence. Where they combine together, they absolutely cut off all hope of improvement, or chance of success.

Wisdom or intelligence seldom comes to us unsought. The Creator has endowed us with an aptitude, a capacity for the acquisition of knowledge, and has thrown around us, in the largest abundance, the materials wherewith to fill that capacity. But still we may not gather up and appropriate to ourselves the rewards and benefits of knowledge, without an effort on our own part. Were we perfect and upright in all respects, and assured against error and depravity, we might sail down the stream of life without thought or danger. But such is not the fact; and while the price of liberty is eternal vigilance, so physical health in general, and sound teeth as well, can only be secured by a careful and constant compliance with the terms on which our physical and physiological conditions are dependent.

